# Dr. Perry H. Millard

**Published:** 1897-03

**Authors:** 


Dr. Perry H. Millard, of St. Paul, Minn., dean of the College of Medicine and Surgery at the State University, and professor of surgery in that institution, died in Johns Hopkins hospital, at Baltimore, February 1, 1897, of pernicious anemia, in the forty-ninth year of his age. He was a native of New York, received his preliminary training at the Ogdensburg Educational Institute, and took his doctorate degree at the Rush Medical College in 1872. After practising medicine several years at Stillwater, Minn., he removed to St. Paul, where he at once took a high position, and continued in active practice until within a few weeks of his death.

Dr. Millard was brought into national prominence a few years ago through his advocacy of a higher standard of medical education, and he became a leader in the contest in Minnesota for a medical practice act, which was adopted as a model by many other states. He was an active organiser and promoter of the American Medical College Association, where again his especial aptitude for leadership in encouraging a higher average standard of medical teaching became apparent. He was also instrumental in organising the national confederation of state medical examining and licensing boards, was chairman of the executive council of that organisation and also chairman of the committee on minimum standard of requirements for admission to practice, which committee is to report at the annual meeting of the confederation to be held at Philadelphia, May 31, 1897.

Dr. Millard was also the principal mover in projecting, organising and developing the department of medicine in the University of Minnesota. He secured the surrender of the charter of private colleges which united in its establishment, and it was through his unceasing labors and persistent enthusiasm that it became a part of the educational system of the state of Minnesota.

The death of Dr. Millard leaves a wide place that is not easy to fill. He was a man useful in so many ways, and, withal, was equipped with so much rare good sense and judgment, which tempered an aggressiveness that never knew anything short of success, that his loss is felt not only in his own city and state, but throughout the union. An appropriate memorial, adopted by the college faculty of which he was a member, sets forth in brief though fitting terms the marvelous aptitude of the man for the places that he occupied, and speaks in tender terms of the widespread affliction that his death has caused.

